# Restoring Severely Atrophic Edentulous Ridge of Mandible Using Self-Expanding Tissue Expander—A Case Report

**DOI:** 10.3390/medicina60050759

**Published:** 2024-05-02

**Authors:** Chiyun Won

**Affiliations:** Purpose Driven Dental Clinic, Seoul 04099, Republic of Korea; wonchiyun@gmail.com

**Keywords:** self inflating tissue expander, bone graft, vertical bone augmentation, posterior mandible

## Abstract

This report describes the use of Self Inflating Tissue Expanders (SITEs) to rehabilitate severely atrophic edentulous mandibular ridges, enabling successful bone grafting and implant placement. The treatment resulted in stable and complication-free implants over a seven-year follow-up, demonstrating SITEs’ effectiveness in providing sufficient bone volume and soft tissue coverage for dental implants.

## 1. Introduction

Bone grafting is a pivotal step in implant restoration, especially when there is insufficient bone quantity [1]. The objectives of bone grafting are threefold: (1) To increase bone volume to accommodate the implant’s minimum size initially, (2) To ensure a bony structure that supports biological width for long-term stability, and (3) To meet esthetic requirements by restoring the balance between living tissue and prosthetic teeth [2].

The desired bone volume for grafting is meticulously planned based on specific objectives. To achieve these goals, a range of bone grafting techniques have been developed, taking into account several crucial aspects:

- Safety: Prioritizing the reduction of surgical risks and complications.
- Efficacy: Focusing on maximizing the graft volume and improving the augmentation of living tissue.
- Longevity: Guaranteeing the graft’s stability over time, preventing any degradation.
- Efficiency: Simplifying the procedure to save time and effort for both the practitioner and patient.
- Patient Comfort: Striving to minimize pain and discomfort during and after the procedure.
- Surgical Sites: Adapting the grafting approach to suit the specific anatomical location.
- Graft Materials: Choosing the most suitable materials for the graft based on the clinical requirements and outcomes desired.

These considerations are essential for the development and selection of bone grafting techniques, ensuring that each approach is tailored to meet the patient’s needs while adhering to basic surgical principles [3].

To achieve these objectives, a variety of techniques are available. These include the Ridge Split technique, Guided Bone Regeneration (GBR) technique, Lateral Sinus Lift and Graft, Transcrestal Approach, Tunnel Technique, Titanium Mesh Technique, Block Bone Graft Technique, Alveolar distraction osteogenesis as well as Simultaneous Grafting with Implant Placement, and Staged Grafting prior to Implant Placement. Additional methods vary according to the graft material used.

It is crucial to employ the most predictable and efficient surgical technique that ensures safety and meets the patient’s needs among the many available options.

Regardless of the chosen bone graft technique, it is imperative to increase soft tissue coverage to achieve tension-free primary closure of the surgical site. This is vital to prevent wound dehiscence, which is a significant risk factor for graft failure or compromised results [4].

Methods to expand soft tissue prior to bone grafting can significantly enhance safety and efficacy. Osmed Company (Ilmenau, Germany) has developed the Self-Inflating Tissue Expander (SITE) [5,6]. designed for use in various surgical applications, including plastic, ophthalmic, and oral surgery. Utilizing the fundamental principles of bone grafting, SITE offers added benefits to the bone graft procedure, particularly in severely atrophic cases [7].

The author has applied SITEs in the bone grafting of severely atrophic mandibular edentulous ridges, with the goal of achieving fixed restoration using implants. The outcomes have been positive, evidenced by a successful follow-up period of 7 years. This case is now presented for review.

The patient provided informed consent for the publication of this case report.

## 2. Case Report

**Figure 1:**
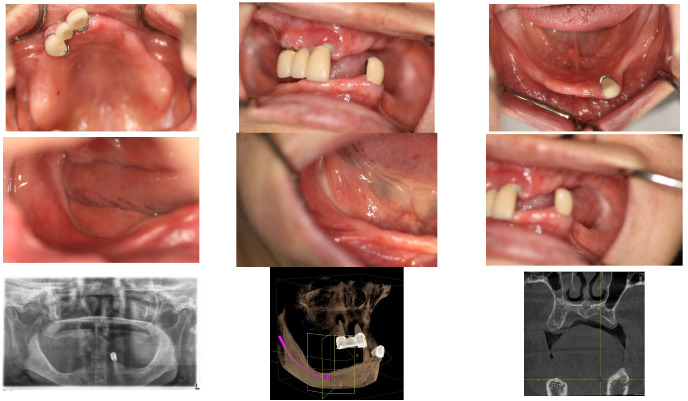
The initial intraoral photos showed that the upper arch had only two teeth remaining, both with root caries and periapical abscesses, while the other teeth were missing. The lower arch displayed severely atrophic mandibular edentulous ridges, with only the left canine remaining. Please note that the residual crest of the right edentulous ridge is flush with the level of the mouth floor.

In April 2015, a 65-year-old female patient sought care at Purpose Driven Dental Clinic, reporting discomfort with her old upper and lower removable dentures. She had no medical history other than controlled hypertension, which was managed with medication. She had her lower posterior teeth extracted decades ago and had undergone several replacements of her removable dentures before visiting my clinic. Examination revealed severely atrophic upper and lower edentulous ridges, save for three remaining teeth (right upper canine, right upper incisor, and left lower canine). Panoramic X-ray imaging indicated that the residual crestal bone height in the first molar area above the upper border of the inferior mandibular canal was 2.2 mm on the right side and 4.0 mm on the left side. The patient expressed her desire for replacing the edentulous areas with fixed prostheses using dental implants (Figure 1).

While dental implant placement was feasible in some upper posterior molar crestal areas without notable challenges, the lower posterior regions lacked sufficient crestal bone for implantation without significant bone augmentation.

Given this requirement, a preparatory bone augmentation procedure was determined to be necessary. However, the soft tissue covering the crestal bone was too scant for effective vertical bone augmentation. Addressing this issue, a Soft Tissue Expansion procedure was undertaken prior to the bone augmentation using a SITE manufactured by Osmed company.

**Figure 2:**
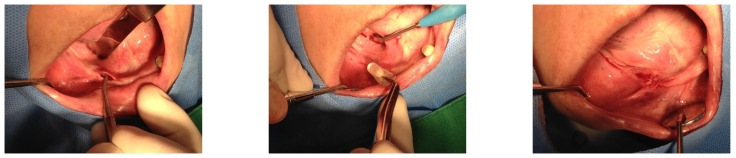
On 28 May 2015, a small incision was made in the right posterior edentulous site of the mandible, through which a Self-Inflating Tissue Expander (SITE) was inserted into a subperiosteal pouch and subsequently sutured. On 2 June 2015, wound dehiscence occurred at the insertion site of the SITE on the right side, necessitating the removal of the SITE. Consequently, a new 0.7 mL SITE was inserted on 9 September 2015.

On 28 May 2015, a 1.3 mL SITE was inserted into the right edentulous ridge through a small pouch created by a 4 mm stab incision. On 2 June 2015, wound dehiscence occurred at the insertion site of the SITE on the right side, necessitating the removal of the SITE. Consequently, a new 0.7 mL SITE was inserted on 9 September 2015 (Figure 2).

**Figure 3:**
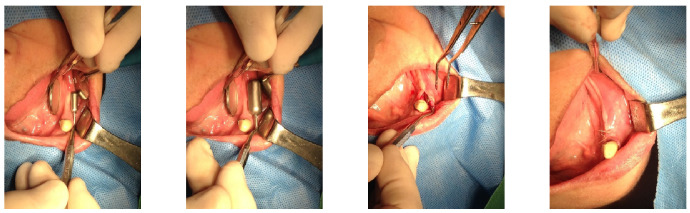
On 1 June 2015, the same procedure was performed at the left posterior edentulous site of the mandible. Please note the use of a before-and-after template for predicting the final result.

Subsequently, on 1 June 2015, the same procedure was performed at the left posterior edentulous site of the mandible and a 1.3 mL SITE (Figure 3).

**Figure 4:**
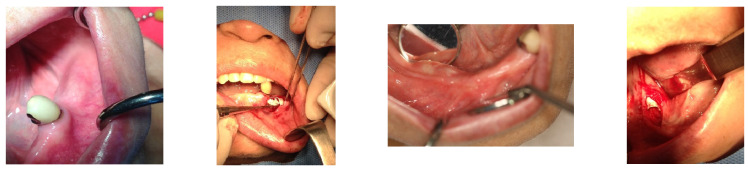
Bone graft procedures were performed when the SITEs were removed after their full expansion. The GBR technique was used with xenograft (Bio-Oss large granule) and Gore-Tex TR membrane. The same small incisions used for removing SITEs on both sides were sufficient for GBR, thanks to the prior soft tissue expansion. The procedures were carried out on different dates for each side, with the left side being treated on 21 August 2015, and the right side on 16 October 2015.

The SITEs were removed after achieving full expansion, in adherence to the manufacturer’s recommendations—82 days later on the left side (21 August 2015) and 37 days later on the right side (16 October 2015). The vertical bone augmentation procedures were conducted simultaneously. Xenograft material (Bio-Oss, produced by Geistlich, Wolhusen, Switzerland). Was utilized for the augmentation and covered with a Goretex titanium-reinforced membrane (produced by W. L. Gore & Associates in the Newark, Delaware, USA) through the same small openings used for the SITEs removal. The incisions were sutured with 4-0 Monocryl, and the stitches were removed two weeks later, revealing no significant wound dehiscence (Figure 4).

On 3 June 2015, in the lower anterior area, implants were placed at the sites of both lateral incisors, measuring 3.6 × 10 mm and 3.6 × 11 mm respectively, using Oneplant Warrentec implants manufactured in Seoul, Rep of Korea. Concurrently, a bone graft was performed using the guided bone regeneration (GBR) technique, involving a collagen membrane from ACE Surgical Supply Co., Brockton, MA, USA, and a xenograft from Bio-Oss, Geistlich, Switzerland, though without the use of a Self-Inflating Tissue Expander (SITE). Importantly, the restoration of this area was completed prior to initiating the implant surgery in the posterior areas.

**Figure 5:**
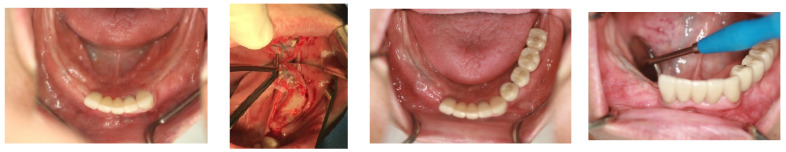
After an 8-month waiting period for bone consolidation, implants were placed in the left posterior area following the removal of the Gore-Tex membrane. It is noteworthy to mention the well-matured bone achieved from the bone graft procedure. Photographs documenting this progression include one from the left side on 1 April 2016, the day the left natural canine crown was set; 18 April 2016, the day of the operation on the left posterior area of the mandible; and two images from the right side on 26 October 2016, the day of implant placements for the right second premolar and the right first molar, 6 months after the second bone graft.

Following an 8-month period for bone consolidation, implants were then placed in the left lower posterior edentulous areas on 18 April 2016. A 4.5 × 9 mm OsseoSpeed TX implant from Astratec, Mölndal, Sweden, was used for the first premolar, and 4.3 × 7 mm and 4.8 × 8 mm Oneplant implants from Warrentec, Seoul, South Korea, were used for the second premolar and the first molar, respectively. Additionally, a 4.0 × 6.0 mm OsseoSpeed TX implant, also from Astratec, Germany, was placed (Figure 5).

Seven months following the first bone graft, performed on the day the SITE was removed from the right posterior area of the mandible, 5 × 9 mm OsseoSpeed TX implants (OsseoSpeed TX implant, Astra Tech, Mölndal, Sweden) were placed for the first premolar and the canine. However, the procedure to place implants in the molar area was deferred; instead, a second bone graft utilizing the GBR technique was conducted to augment the bone volume, ensuring it could accommodate implants of the appropriate size.

**Figure 6:**
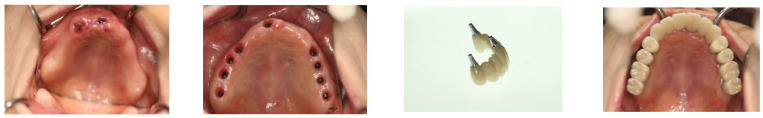
The fixed restorations with implants for the upper arch were completed more simply than those for the lower arch because the existing bone had sufficient quantity to support the implants. The prostheses were divided into five pieces: one anterior bridge, two premolar single crowns, and two bridges for the molar areas. To simplify the procedure and facilitate ease, the most favorable sites were selected for anterior implant placement. Please note the path for inserting the anterior implants, which includes one path extraorally after cementation.

In the upper jaw, implants (Oneplant Warrentec, manufactured in Seoul South Korea) were inserted using standard techniques, selecting the most advantageous sites for implant placement for the patient’s convenience. The final fixed prosthodontics consisted of five pieces: an anterior piece spanning from the right premolar to the left premolar, both second premolars, and a two-unit posterior molar bridge on each side. All implant prostheses were cemented with ready-made stock abutments extraorally and secured by screw tightening, completing all procedures on 27 January 2016 (Figure 6).

**Figure 7:**
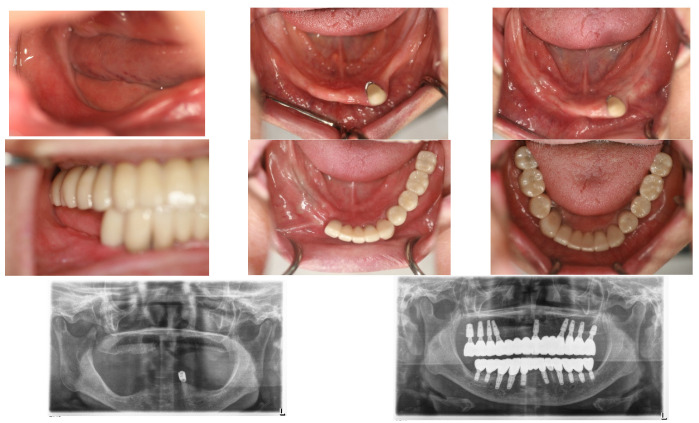
On 2 May 2017, the fixed restorations with implants for the lower arch were completed, 23 months after the commencement of the procedure, through vertical bone grafting via SITE. The completion on the right side was delayed compared to the left side due to the need for more bone regeneration, although both sides underwent the same procedure. Please compare the before-and-after treatment pictures.

The implant restorations for the lower left posterior area were finalized on 28 September 2016, five months following the implant placements. Subsequently, the restorations for the lower right canine and first premolar were completed on 23 December 2016, seven months post-implantation. The final restorations for the lower right first molar and second molar were accomplished on 2 May 2017, six months after their implantation. This marked the conclusion of all fixed prosthesis work, spanning a total of 23 months from the beginning to the end of the treatment process (Figure 7).

**Figure 8:**
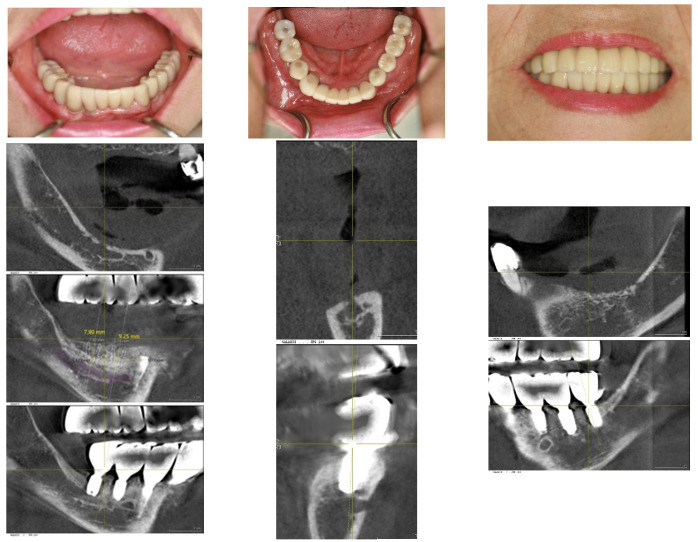
After 5 months, on 24 October 2017, a follow-up visit confirmed the success of the treatments. The soft tissue displayed healthy architecture without compromising the vestibular depth. The proportion between the crown height and the mucosal portion appeared natural. Notably, there were no soft tissue complications, such as bleeding, swelling, or erythema. From the frontal view, the overall appearance was deemed acceptable.

Throughout all procedures, the patient tolerated the treatments well, experiencing no surgical complications such as nerve injury or wound infection. She used temporary removable dentures; however, during the critical postoperative period, which lasted about 2 months, she was advised against wearing them to avoid potential surgical complications from trauma. After this critical period, she was allowed to wear the temporary removable dentures, provided they were adjusted to alleviate pressure on the surgical site. The patient’s chewing ability was effectively restored, with occlusion being verified using Shimstock. The graft bone demonstrated stable results, showing no signs of inflammation or resorption. The implants were functioning well, with no mobility or pain reported. The patient expressed satisfaction with the outcome (Figure 8).

During the follow-up period extending to 2024, a minor prosthetic complication occurred: the upper anterior bridge became detached due to the failure of the dental cement, resulting in a loss of retention between the bridge and the implant abutment. Additionally, a small fracture was noted in a portion of the marginal area, characterized by chipping. The patient opted against the fabrication of a new bridge, preferring instead to have the existing anterior bridge re-cemented. This repair was straightforward and did not necessitate the creation of a new prosthetic.

Except for this event, there have been no other complications in both biological and mechanical aspects. This means there have been neither peri-implant mucositis nor peri-implantitis, and there have been no prosthodontic complications.

**Figure 9:**
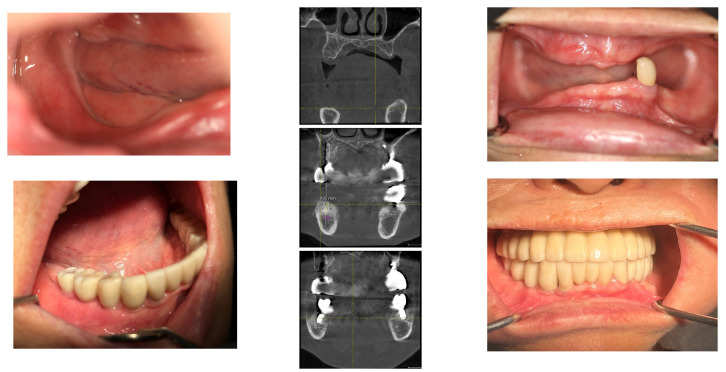
The lower pictures were taken on 2 May 2023, and 9 January 2024, each six years and seven years after the final finishing, respectively. Please compare them with the initial pictures taken before treatment. The rehabilitation using SITE has shown stable results throughout the follow-up period.

The patient’s masticatory function has been almost fully restored, without any discomfort or difficulties during eating. Her appearance looks natural, as though she had her natural teeth, even though she does not (Figure 9).

## 3. Results

Vertical bone changes at the right operation site were evaluated before surgery and then annually post-surgery for comparison. Measurements were taken from the superior border of the inferior alveolar canal to the crest of the ridge at two designated points: Point A, the lowest part of the edentulous ridge at baseline, and Point B, positioned above the mental foramen. To ensure measurement consistency, the length of the right upper second molar implant was used as a calibration standard. Prior to these measurements, to ascertain the repeatability of the points A and B assessments, the rectangular distance from the inferior border of the inferior alveolar canal to the bottom border of the mandible was verified.

**Figure 10:**
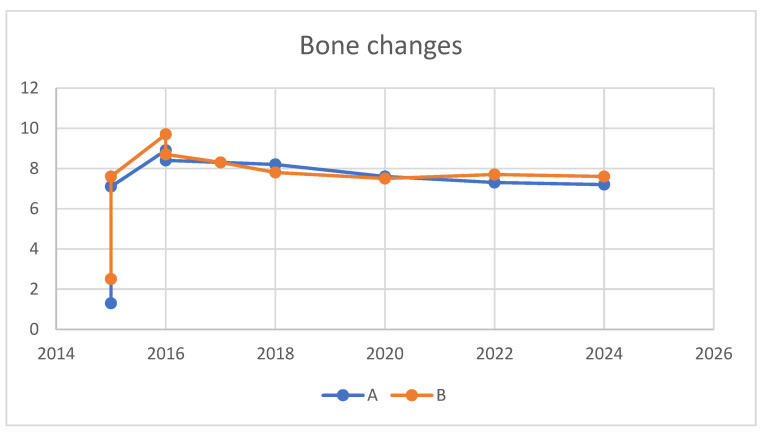
This graph illustrates the vertical bone changes at the right operation site, detailing measurements from Point A—the baseline’s lowest part of the edentulous ridge—and Point B, which is located above the mental foramen.

**Figure 11:**
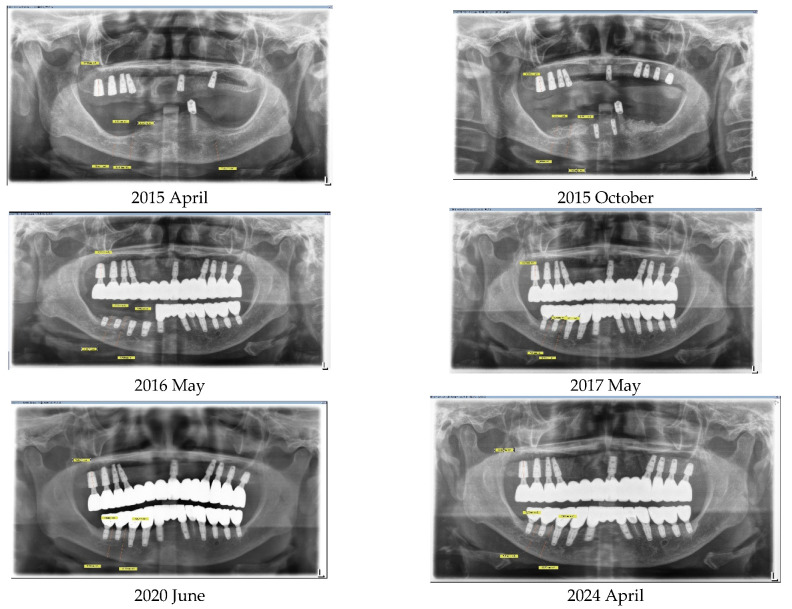
Panoramic X-ray images displaying the perioperative state along with the annual changes postoperatively.

**Figure 12:**
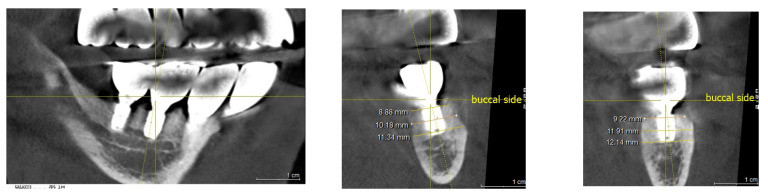
Illustrating the horizontal width measurements at the crestal level, showing 8.9 mm at Point A and 9.2 mm at Point B. Further down, at the middle level, the measurements increased to 10.1 mm and 11.9 mm, respectively, demonstrating a sufficient bone width necessary for implant support.

Initially, in April 2015, the heights at Points A and B were recorded as 1.3 mm and 2.5 mm, respectively. Subsequent to the first bone graft, facilitated by SITE (Self Inflating Tissue Expander), the heights increased to 7.1 mm at Point A and 7.6 mm at Point B by October 2015, resulting in vertical increments of 5.8 mm at Point A and 5.1 mm at Point B. Following the second bone graft, minor increases were observed in May 2016, with heights reaching 8.9 mm at Point A and 9.7 mm at Point B. By the day of implant prosthesis completion in May 2017, both Points A and B recorded a crestal height of 8.3 mm. These heights were then tracked annually, showing a marginal decrease of 0.1 mm at point B and 0.15 mm at point A from the prosthesis completion in May 2017 to 2024. Over a span of seven years, the overall decreases were 1.1 mm at Point A and 0.7 mm at Point B, deeming these results indicative of stable crestal bone changes (Figure 10, Figure 11 and Figure 12).

Table 1 summarize a comprehensive overview of the crestal height changes at Points A and B from the initial measurement in April 2015, through the interventions and up to the most recent check in June 2024.

## 4. Discussion

Bone grafting has emerged as a cornerstone in the rehabilitation of oral tissues lost to various causes, including teeth, bone, and the surrounding soft tissues, through dental implantation [8,9]. The loss of teeth invariably leads to the concurrent loss of interdependent tissues, underscoring the complexity of oral rehabilitation [10]. Although implant dentistry primarily aims to restore chewing function, it also encompasses the regeneration of all missing oral tissues, regardless of their immediate contribution to functional improvement.

Bone grafting is crucial for implantation under two primary circumstances. Firstly, it is necessary when the available sites for implantation lack sufficient volume to support the smallest implant sizes, rendering the restoration unfeasible without further augmentation [3]. Secondly, aesthetic considerations and the aim for lasting stability come into play, notably in restoring anterior teeth. This approach is equally important for posterior teeth restorations [9]. For example, a clinical crown that is excessively long compared to adjacent teeth may look unnatural. Additionally, an optimal bone phenotype around the implants, characterized by a proper bone thickness, plays a vital role in maintaining their biological stability over time. Both circumstances, alongside the established understanding that tooth loss inevitably leads to the simultaneous loss of bone and soft tissues, highlight the fundamental importance of bone grafting in implant dentistry.

A broad spectrum of techniques is employed in bone grafting for dental implants, designed to tackle the varied clinical scenarios encountered in patient care. These procedures can be categorized based on the recipient site’s topography into two main types: grafts for internal contained defects and grafts for external augmentation. The former includes methods such as socket preservation and sinus grafts, which are directed at repairing deficits within the bone structure. Meanwhile, external augmentation often incorporates a soft tissue releasing procedure integral to the bone grafting process itself, underlining the diversity and complexity of approaches needed to satisfy the comprehensive range of anatomical and aesthetic requirements. However, the pivotal element in external augmentation lies in flap management, which includes soft tissue expansion to ensure primary closure and prevent wound dehiscence, further emphasizing the critical role of meticulous soft tissue handling in the success of these procedures [11].

From this perspective, the soft tissue expander was conceived. Throughout the development process for the right device, Osmed company, based in Germany, introduced the SITE, a notable innovation in this field. The Osmed tissue expander is made of a hydrogel material. Hydrogels are highly absorbent polymers that can retain a significant amount of water or biological fluids, swelling to several times their original size. This property allows the Osmed tissue expanders to gradually expand as they absorb bodily fluids, thereby stretching the surrounding tissues over time. The specific composition of the hydrogel used in Osmed tissue expanders is designed to ensure biocompatibility, optimal expansion rates, and sufficient mechanical strength to meet the requirements of the intended medical or dental applications [5,6,7].

If the required volume for external bone augmentation for implant placement is moderate or less, the bone grafting can be performed with conventional techniques, accompanied by soft tissue releasing. However, cases that demand a significantly larger volume for augmentation pose considerably greater challenges [11,12].

While other alternative techniques could have been considered [13], the author opted to utilize the SITE for the case presented.

Among various alternative techniques available for consideration, the decision to utilize the SITE for this case was driven by its unique benefits that specifically addressed the clinical challenges encountered.
Advantages of SITE: 1. Increased Soft Tissue Volume: SITE can increase soft tissue volume in advance, minimizing the risk of wound dehiscence that could deteriorate the outcomes of bone graft procedures. 2. Vestibule Depth Maintenance: Unlike traditional bone graft procedures, which sometimes compromise vestibule depth due to the required releasing procedures, SITE maintains this critical anatomical feature. 3. Handling Complex Cases: SITE enables specialists to manage extremely difficult cases, achieving optimal results that might be unattainable without this technology.Disadvantages of SITE: 1. Additional Costs: The use of SITE incurs extra expenses, both in terms of time and financial investment. 2. Special Skills Required: Effective management of SITE requires specialized skills, adding a level of complexity to treatment.


The initial attempt to insert a SITE into the right posterior area of the mandible was unsuccessful due to wound opening at the insertion entrance, necessitating the SITE’s removal. This issue highlights a fundamental flaw in the design of the Osmed SITE, particularly the envelope area designed for securing the SITE to the underlying bone. The extension of this area, which caused irritation at the entrance site, should have been positioned deeper. This inherent drawback became a valuable lesson from which I learned to avoid repeating the same mistake in subsequent procedures.

The upper arch (full edentulous arch) was successfully restored using standard dental implant procedures without any significant issues. However, a specific treatment plan was devised for the upper side, focusing on selecting the optimal locations for implant placement to achieve the desired outcomes more easily and efficiently. Thus, for the anterior part of the upper arch, the sites for both first premolars and the left incisor were chosen for an 8-unit implant bridge. The implants were positioned in parallel to ensure that the prosthesis could be securely attached by screwing it in place after extraoral cementation, with the margins positioned subgingivally to fulfill aesthetic requirements.

The lower arch, which was fully edentulous except for the left canine, was segmented into four parts to facilitate the surgical process in stages.

The right and left posterior edentulous ridges were severely atrophic, resulting in a minimal distance between the ridge crest and the inferior alveolar canal, alongside a very narrow crest width. Consequently, the majority of the alveolar bone needed for implant placement had to be regenerated through bone grafting to ensure successful outcomes. This was particularly critical for the right posterior ridge, where virtually no bone was available for implant treatment. Initially, the distance measured 1.5 mm at Point A and 2.3 mm at Point B, with Point A representing the lowest part of the edentulous ridge at baseline and Point B located above the mental foramen. Ultimately, as detailed earlier in this report’s case study, the bone grafting procedure using the Self-Inflating Tissue Expander (SITE) proved successful for implant placement. The maximum amount of vertical bone gain achieved was 7.6 mm at Point A and 7.2 mm at Point B.

The horizontal width measurements at the crestal level showed 8.9 mm at Point A and 9.2 mm at Point B. Deeper, at the middle level, the measurements increased to 10.1 mm and 11.9 mm, respectively, indicating a substantial growth in bone width crucial for implant support. The peak horizontal bone augmentation reached was 11.9 mm, signifying notable enhancements in the dimensions vital for successful implant placement (Figure 12).

The final outcome with the implant prostheses was realized in May 2017, after nearly two years of treatment from the onset. The patient has undergone annual follow-ups since completion of the prosthesis. Crestal bone loss has been recorded at a rate of 0.1 mm per year at point B and 0.15 mm per year at point A, which is deemed stable [14]. Throughout this period, there have been no specific events or complications related to the implants, either surgically or prosthodontically. For the left side, similarly excellent results have been observed, with no more than 0.5 mm of bone loss recorded up to 2024 since the treatment’s completion.

Upon deeming the bone graft procedure successful, the following claim is substantiated: After achieving pre-surgical objectives and ensuring stable outcomes from bone grafts, it’s vital to recognize that the determinants of implants’ long-term success do not differ, regardless of bone grafting. These determinants, affecting both the biological and mechanical integrity of implants, can lead to issues like peri-implantitis and mechanical problems such as abutment fractures, impacting implant performance equally in grafted and non-grafted situations. Thus, the use of a bone graft does not modify the risk factors for peri-implantitis [15,16].

Concerning the selection of graft material, Bio-Oss xenograft from Geistlich Pharma was exclusively utilized [17,18,19]. The outcomes of this report confirm that the graft material’s function, acting solely as a scaffold, is effectively achieved, facilitating the regeneration of new bone as long as space is maintained for the bone to form [20]. Notably, this space was established using the SITE method, avoiding typical surgical complications such as wound dehiscence.

## 5. Conclusions

The SITE played a pivotal role in the successful restoration of an extremely atrophic mandibular edentulous case with fixed dental implants. The space created by SITE enabled successful regeneration without the need for an autogenous bone graft. This technique facilitated a maximum vertical gain of 7.6 mm. Follow-up results over seven years have demonstrated stability in the outcomes, with minimal crestal bone loss recorded at 0.1 mm per year at point B and 0.15 mm per year at point A per year, underscoring the effectiveness and durability of the SITE approach in challenging dental restorations.

## Figures and Tables

**Table 1:** Showing a comprehensive overview of the crestal height changes at Points A and B from the initial measurement in April 2015, through the interventions and up to the most recent check in June 2024. The notes column provides additional context to the measurements, highlighting the significant gains after the grafts and the slight annual decreases post-implant completion, which have been deemed stable.

Time	Point A Height (mm)	Point B Height (mm)	Notes
April 2015	1.3	2.5	Initial Measurements
October 2015	7.1	7.6	After 1st Bone Graft (Vertical Gains: 5.8 mm & 5.1 mm for A & B)
May 2016	8.9	9.7	After 2nd Bone Graft (Increases: 1.8 mm & 2.1 mm for A & B)
October 2016	8.4	8.7	Adjustment Post-2nd Graft
May 2017	8.3	8.3	At Completion of Implant Prostheses
April 2018	8.2	7.8	Annual Check
June 2020	7.6	7.5	Annual Check
December 2022	7.3	7.7	Annual Check
June 2024	7.2	7.6	Latest Measurement (Total Reductions: 1.1 mm & 0.7 mm for A & B over 7 years)

## Data Availability

All data supporting the findings of this study were generated in-house. Measurements were conducted personally as part of the research process. The images included in the manuscript serve as primary evidence supporting the data and results presented. Currently, the data are not publicly archived but are available from me, the corresponding author, upon reasonable request.

## References

[1] Dimitriou R., Jones E., McGonagle D., Giannoudis P.V. (2011). Bone regeneration: Current concepts and future directions. BMC Med..

[2] Wang C.I., Barootchi S., Tavelli L., Wang H. (2021). The peri-implant phenotype and implant esthetic complications. Contemporary overview. J. Esthet. Restor. Dent..

[3] Buser D., Urban I., Monje A., Kunrath M.F., Dahlin C. (2023). Guided bone regeneration in implant dentistry: Basic principle, progress over 35 years, and recent research activities. Periodontology 2000.

[4] Garcia J., Dodge A., Luepke P., Wang H., Kapila Y., Lin G. (2018). Effect of membrane exposure on guided bone regeneration: A systematic review and meta-analysis. Clin. Oral Implant. Res..

[5] Obdeijn M.C., Nicolai J.-P.A., Werker P.M. (2009). The osmotic tissue expander: A three-year clinical experience. J. Plast. Reconstr. Aesthetic Surg..

[6] Uijlenbroek H.J., Liu Y., He J.F., Visscher C., van Waas M.A., Wismeyer D. (2011). Expanding soft tissue with Osmed tissue expanders in the goat maxilla. Clin. Oral. Implants Res..

[7] Mertens C., Thiele O., Engel M., Seeberger R., Hoffmann J., Freier K. (2015). The use of self-inflating soft tissue expanders prior to bone augmentation of atrophied alveolar ridges. Clin. Implant. Dent. Relat. Res..

[8] Cha H.-S., Kim J.-W., Hwang J.-H., Ahn K.-M. (2016). Frequency of bone graft in implant surgery. Maxillofac. Plast. Reconstr. Surg..

[9] Le B., Hayashi N. (2022). The Aesthetic Contour Graft—Enhancing peri-implant soft tissue contours and pontic sites with guided bone regeneration. J. Esthet. Restor. Dent..

[10] Bodic F., Hamel L., Lerouxel E., Baslé M.F., Chappard D. (2005). Bone loss and teeth. Jt. Bone Spine.

[11] Urban I., Monje A., Lozada J., Wang H.-L. (2017). Principles for Vertical Ridge Augmentation in the Atrophic Posterior Mandible: A Technical Review. Int. J. Periodontics Restor. Dent..

[12] Urban I.A., Montero E., Amerio E., Palombo D., Monje A. (2023). Techniques on vertical ridge augmentation: Indications and effectiveness. Periodontology 2000.

[13] Andre A., Ogle O.E. (2021). Vertical and Horizontal Augmentation of Deficient Maxilla and Mandible for Implant Placement. Dent. Clin. N. Am..

[14] Demenko V., Linetskiy I., Linetska L., Nesvit V., Shevchenko A., Yefremov O., Weisskircher H.-W. (2016). Prognosis of implant longevity in terms of annual bone loss: A methodological finite element study. Comput. Methods Biomech. Biomed. Eng..

[15] Al-Abedalla K., Torres J., Cortes A.R.G., Wu X., Nader S.A., Daniel N., Tamimi F. (2015). Bone Augmented with Allograft Onlays for Implant Placement Could Be Comparable with Native Bone. J. Oral Maxillofac. Surg..

[16] Benić G.I., Jung R.E., Siegenthaler D.W., Hämmerle C.H.F. (2009). Clinical and radiographic comparison of implants in regenerated or native bone: 5-year results. Clin. Oral Implant. Res..

[17] Goyal S., Masood M., Le C., Rajendran Y., Nanjapa S., Vaderhobli R. (2021). Comparative Bone Graft Evaluation for Dental Implant Success: An Evidence-Based Review. J. Long Term Eff. Med. Implant..

[18] Sheikh Z., Hamdan N., Ikeda Y., Grynpas M., Ganss B., Glogauer M. (2017). Natural graft tissues and synthetic biomaterials for periodontal and alveolar bone reconstructive applications: A review. Biomater. Res..

[19] Majzoub J., Ravidà A., Starch-Jensen T., Tattan M., Del Amo F.S.-L. (2019). The Influence of Different Grafting Materials on Alveolar Ridge Preservation: A Systematic Review. J. Oral Maxillofac. Res..

[20] Abu-Mostafa N.A., Alotaibi Y.N., Alkahtani R.N., Almutairi F.K., Alfaifi A.A., Alshahrani O.D. (2022). The Outcomes of Vertical Alveolar Bone Augmentation by Guided Bone Regeneration with Titanium Mesh: A Systematic Review. J. Contemp. Dent. Pract..

